# Climate change perspectives in Lithuania: exploring science attitudes and personality correlations

**DOI:** 10.3389/fpsyg.2026.1696411

**Published:** 2026-04-02

**Authors:** Aelita Skarzauskiene, Monika Mačiulienė, Aistė Diržytė

**Affiliations:** 1 Mykolas Romeris University, Vilnius, Lithuania; 2 Vilnius Gediminas Technical University, Vilnius, Lithuania

**Keywords:** attitudes toward science, climate change, latent profile analysis, Lithuania, science communication

## Abstract

**Introduction:**

Public responses to climate change are influenced by interpretations of scientific information and individual differences. Understanding these factors can improve targeted climate communication.

**Methods:**

We conducted a nationally representative survey of Lithuanian adults (*N* = 1,005; fieldwork 21 June–7 July 2024) to examine (a) distinct profiles of climate-change beliefs, (b) the science attitudes differentiating these profiles, and (c) whether personality traits relate to climate-change attitudes directly and indirectly via science attitudes. Latent profile analysis (LPA) identified belief profiles, binary logistic regression assessed predictors of profile membership, and mediation analyses tested indirect effects through attitudes toward science.

**Results:**

LPA using three climate-belief indicators supported a two-class solution among respondents with complete data (*n* = 930): Lower Endorsement (29.2%) and Higher Endorsement (70.8%). Stronger endorsement of science’s problem-solving capacity, support for unrestricted scientific inquiry, and support for state funding of research significantly increased the odds of belonging to the Higher Endorsement class (*n* = 652 with complete predictor data). Mediation analyses indicated that science attitudes were positively associated with climate-change attitudes and partially mediated the relationships between conscientiousness, extraversion, neuroticism, and climate-change attitudes.

**Discussion:**

The findings reveal heterogeneity in climate-change beliefs in Lithuania and suggest that audience segmentation and psychologically informed communication strategies may enhance climate-related science communication.

## 1 Introduction

Science communication is central to public engagement with complex societal risks such as climate change because it shapes how people notice, evaluate, trust, and use scientific information. Public audiences, however, are heterogeneous: individuals differ in their information attention, epistemic judgments, and trust in institutions and experts, and these differences often consolidate into segments with distinct informational needs and motivational barriers. Such heterogeneity is especially consequential for contested issues like climate change, where skepticism, misinformation, and issue fatigue can weaken engagement and polarize public discussion (Nisbet et al., 2015; Mede et al., 2022; Klinger et al., 2022a, 2022b). Accordingly, climate communication benefits from identifying groups that are skeptical or disengaged and clarifying the psychological factors that differentiate them. Audience segmentation research consistently shows that individuals cluster into groups characterized by distinct constellations of beliefs, information practices, and motivations, implying that “one-size-fits-all” messaging is unlikely to be effective (Metag and Schäfer, 2018; Scheufele, 2018; Schäfer et al., 2018).

Recent climate communication research indicates that engagement is filtered through attention, identity, emotions, and trust in science and scientific actors (Leiserowitz et al., 2021; Cologna et al., 2024; Hutmacher et al., 2024; Bird et al., 2024). Large-scale segmentation studies show that publics cluster into qualitatively distinct groups defined by configurations of climate beliefs, perceived risks, policy preferences, and behavioral intentions; importantly, polarization may emerge even within an overall consensus, producing subgroups that agree climate change is real but diverge sharply on urgency, acceptable solutions, or trust in institutions, thereby undermining generic communication campaigns (Leiserowitz et al., 2021; Kácha et al., 2022; Klinger et al., 2022a, 2022b; Lind et al., 2023; Reif et al., 2025). Trust is a particularly decisive differentiator: trust in climate science and climate scientists is consistently linked to greater acceptance of anthropogenic climate change and stronger support for mitigation and adaptation, whereas distrust amplifies susceptibility to misleading narratives and reduces willingness to treat scientific claims as action-relevant (Cologna et al., 2024). Moreover, climate misinformation and disinformation have become durable features of many media ecosystems. Experimental evidence suggests that both pre-bunking (inoculation) and debunking (corrections) can reduce misperceptions, although effects vary by audience characteristics, including predispositions and political attitudes (Bogert et al., 2024; Christner et al., 2024; van Stekelenburg et al., 2022). These dynamics help explain why skepticism and disengagement are not simply functions of “low knowledge,” but often reflect motivated reasoning, identity-protective cognition, and differences in trust calibration (Hutmacher et al., 2024).

Lithuania provides a valuable and theoretically informative setting for climate-related science communication research because climate policy, energy security, and broader geopolitical concerns co-exist, potentially shaping issue salience and trust in institutions. Recent European surveys indicate that Lithuanians generally recognize the need to adapt to climate change and report substantial exposure to extreme weather events, while simultaneously ranking other societal priorities high, which can influence how climate messages are received (European Investment Bank, 2024; European Parliament Research Service, 2025). Representative Lithuanian survey evidence also suggests that climate perceptions and perceived acceptability/risks of energy technologies are meaningfully patterned by environmental worldviews and identity-related orientations, while broader energy concerns may compete with climate priorities in public judgment (Balžekienė and Budžytė, 2021). In parallel, Lithuania’s evolving energy-security transition highlights how socio-economic perceptions and policy contexts can shape public concern, making it especially important to understand which subgroups are skeptical or disengaged and why (Genys et al., 2024). These contextual features strengthen the rationale for applying segmentation methods that can identify distinct climate-belief profiles and connect them to psychological correlates.

The broader science communication literature likewise emphasizes that science communication is not limited to knowledge transmission but also involves engagement and dialogue that should be adapted to the needs of different audiences (Lewenstein, 2022). Besley (2018) argues that science communication audiences range from the scientifically literate to the disengaged and include diverse stakeholders such as policymakers, industry representatives, educators, and the general public; audiences can be categorized by their interest, knowledge, and degree of engagement, and effective strategies should reflect these differences. Consistent with this perspective, Metag and Schäfer (2018) highlight recurring audience types. The Engaged Public tends to show high interest, actively seek science information, and be motivated by curiosity and learning (Metag and Schäfer, 2018). The Interested but Passive audience shows moderate interest and is more likely to engage when science communication is accessible and relevant to everyday life; narratives, relatable stories, and practical examples may increase engagement in this group (Bilandzic et al., 2020; Dudley et al., 2023). The Disengaged Public typically has low interest and limited knowledge, and targeted outreach and co-creation approaches have been recommended to build relevance and reduce barriers (Humm and Schrögel, 2020; Humm et al., 2020). The Skeptical or Critical Public may distrust science or hold negative attitudes toward scientific topics; Nisbet et al. (2015) emphasize that addressing this group requires acknowledging concerns, building trust, and using values-based communication that resonates with worldviews. Finally, Underserved Audiences include marginalized groups who face cultural, socio-economic, or educational barriers to accessing scientific information; inclusive, equity-oriented strategies are crucial for reaching these audiences (Judd and McKinnon, 2021; Humm et al., 2020). Methodologically, segmentation has been pursued through quantitative and qualitative approaches: Ong et al. (2023) used latent profile analysis to identify segments based on risk perceptions and preventive behaviors during the COVID-19 pandemic, illustrating the broader applicability of person-centered segmentation to science communication contexts. Qualitative methods such as focus groups and interviews are particularly useful for understanding underserved or skeptical audiences and for co-creating content with communities (Burns and Medvecky, 2018; Humm et al., 2020), whereas social media analytics can illuminate how different segments engage with science content online (Martin and MacDonald, 2020).

In parallel, recent scholarships have emphasized evidence-based and participatory approaches to science communication. Jensen and Gerber (2019) argue that evidence-based science communication is necessary for determining what works and what does not when engaging diverse audiences. Fischhoff (2019) similarly stresses the importance of evaluating science communication efforts to improve effectiveness. Kessler et al. (2022) examined how academics understand and practice science communication across contexts, reinforcing the value of evidence-based practices that align with the needs and expectations of different audience segments. Participatory approaches, including citizen science, have been argued to help bridge gaps between scientists and publics (Giardullo et al., 2023). Wilkinson et al. (2022) emphasize that participatory science communication requires attention to the roles, incentives, and training needs of those involved, while Hu et al. (2022) note that motivation to participate in secondary science communication is often driven by the desire to contribute to societal change, consistent with sustainable development goals. Despite this extensive literature on science communication audience types and strategies, audience segments defined specifically by climate-change attitudes and their links to attitudes toward science remain under-explored, including in Lithuania.

A further underdeveloped area concerns the role of personality traits. Personality traits capture relatively stable tendencies in emotion, motivation, and social behavior that can influence how people process information and respond to persuasive messages. Prior work suggests that traits such as conscientiousness, extraversion, and neuroticism may be associated with information seeking, social engagement, and sensitivity to threat or risk, which can translate into differences in science-related attitudes and climate engagement. Recent meta-analytic and longitudinal evidence links traits, especially openness, agreeableness, and conscientiousness, to climate denial, concern, knowledge, and pro-environmental behavior, suggesting that personality may contribute to audience segmentation and to the psychological pathways connecting science attitudes with climate engagement (Cipriani et al., 2024; Freminot et al., 2024; Tucholska et al., 2024). At the level of communication strategy, Hirsh et al. (2012) argue that personality traits influence how individuals respond to scientific information, making established psychometric tools such as the Big Five Inventory potentially useful for psychologically informed audience segmentation. Hirsh et al. (2012) propose that persuasive appeals may be more effective when matched to personality, for example, openness may facilitate receptivity to novel and abstract concepts, whereas conscientiousness may align with preferences for practical and actionable information. Complementing this perspective, Lynnyk (2023) discusses the application of personality archetypes in branding, and Olsacher et al. (2023) provide an example of personality-tailored messages on social media to increase engagement; for instance, community-benefit frames may be more persuasive for individuals high in agreeableness, whereas achievement-oriented frames may appeal more to highly extraverted individuals. Nonetheless, the links between personality traits and attitudes toward climate change and science remain under-explored in nationally representative Lithuanian samples.

Therefore, the present study applies a person-centered segmentation approach, latent profile analysis (LPA), to identify distinct climate-belief profiles in a representative Lithuanian sample and to examine how these profiles relate to attitudes toward science and personality traits.

The study addressed three research questions: (RQ1) How many audience segments (latent profiles) can be identified based on climate-change belief indicators in a representative Lithuanian sample? (RQ2) Which science attitudes significantly predict membership in the identified climate-belief profiles? (RQ3) How are personality traits related to attitudes toward climate change and attitudes toward science, including indirect (mediated) associations?

## 2 Methods

### 2.1 Design

We conducted a cross-sectional, nationally representative survey of Lithuanian residents aged 18 years and older. Fieldwork was carried out from 21 June to 7 July 2024 by the Lithuanian–British market and public opinion research company “Baltijos tyrimai.” Interviews were administered in person using a standardized questionnaire (individual interviews at respondents’ residences).

### 2.2 Sampling

Participants were selected using multi-stage stratified random sampling consistent with ESOMAR/European survey practices. At stage 1, the number of interviews per county was proportional to the county’s share of the national adult population. At stage 2, within each county, interviews were allocated proportionally across settlement-size strata. At stage 3, specific sample points (settlements) were selected at random within strata. Within sample points, households were approached using a random-route procedure; within households, one eligible adult was selected using the nearest-birthday rule. If a selected household or respondent was unavailable, interviewers made up to three contact attempts (visits) at different times/days before substituting according to the route protocol. Inclusion criteria were: (a) residence in Lithuania, (b) age ≥18 years, and (c) ability to complete the interview in Lithuanian as offered by the survey agency. Commonly excluded populations in household surveys (e.g., institutionalized individuals such as prisoners and inpatients, and persons without a fixed residence) were not sampled.

### 2.3 Participant characteristics

A total of *N* = 1,005 interviews were completed across 112 sample points (30 towns/cities and 42 rural locations). Sociodemographic characteristics are shown in Table 1.

**Table 1:** Sociodemographic characteristics of participants.

Characteristic	*n*	%
Gender		
Men	459	46
Women	546	54
Age (years)		
18–29	149	15
30–49	340	34
≥50	516	51
Nationality		
Lithuanian	917	91
Polish	40	4
Russian	27	3
Other	14	1.4
No answer	6	0.6
Monthly household income (EUR)		
≤1,200	265	27
1,201–2000	283	28
>2000	213	21
Do not know/no answer	245	24
Education		
University (MA/PhD)	52	5
University (BA)	113	11
College (non-university HE)	115	12
Technical/higher vocational	181	18
Professional	303	30
Secondary	182	18
Primary/incomplete secondary	59	6
Settlement type		
Major cities (Vilnius, Kaunas, Klaipėda, Šiauliai, Panevėžys)	429	43
Other cities/towns	258	25
Rural areas (<2000 inhabitants)	318	32

### 2.4 Ethics

The study was conducted in accordance with Mykolas Romeris University ethical guidelines and was approved by the relevant institutional ethics review board (protocol No. 10-528). Participation was voluntary. The survey was anonymous: no directly identifying information (e.g., names, personal codes, exact addresses) was collected, and results are reported only in aggregate.

### 2.5 Measures

The research questionnaire was created based on Switzerland science audience clustering instrument (Schäfer et al., 2018), attitudes toward fake news analysing tool, used by sample research in Germany (Reuter et al., 2020) and personality traits instrument Big Five 2 adapted for this research (Soto and John, 2017; Sindermann et al., 2020; Calvillo et al., 2021).

Table 2 summarizes the key measures used in the analyses. Items were rated on a 5-point Likert-type scale (1 = strongly disagree to 5 = strongly agree). Composite variables were computed as the mean of their items, with higher scores indicating stronger endorsement.

**Table 2:** Summary of key study measures and response options.

Construct	Source/description	Items used in this study	Response scale	Scoring
Climate-change beliefs (LPA indicators)	Adapted from prior climate attitude items used in audience segmentation surveys	(1) “Climate change is real”; (2) “Climate change is mainly caused by human activity”; (3) “Climate change consequences will be dangerous.”	1–5 Likert	Used as three separate indicators in LPA; composite used for reliability/descriptives
Science-belief predictors (logistic regression)	Adapted from science attitudes/audience segmentation instruments (Schäfer et al., 2018)	Science can solve any problem; Science improves people’s lives; No limits on what can be studied; Research is necessary even if not immediately applicable; Research should be funded by the state; Political decisions should be based on scientific findings; Benefits of science outweigh negative effects.	1–5 Likert	Items entered simultaneously as predictors
Attitudes toward science (mediator; composite)	Adapted from science attitudes/audience segmentation instruments (Schäfer et al., 2018)	Seven science-belief items (listed above)	1–5 Likert	Mean of items (higher = more positive attitudes toward science)
Personality traits	BFI-2-XS short form (Soto and John, 2017)	Three-domain scores retained (neuroticism, extraversion, and conscientiousness) due to acceptable internal consistency; open-mindedness and agreeableness were excluded.	1–5 Likert	Mean of the three items per domain

### 2.6 Data preparation and missing data

Analyses were conducted on all available cases for each analytic step. For LPA, respondents with missing data on any of the three climate-belief indicators were excluded (complete-case analysis), resulting in *n* = 930. For logistic regression, listwise deletion on the seven science-belief predictors and the LPA class variable yielded *n* = 652. For bivariate descriptives/correlations, sample sizes varied slightly by pairwise availability. The patterns of missingness primarily reflected item non-response (e.g., “do not know”/no answer on income or attitudinal items) rather than systematic drop-out.

### 2.7 Statistical analyses

SPSS, JASP, and JAMOVI software were used to analyse the data. Initially, the reliability of the instruments used in this study and the descriptives were calculated. Afterward, the Latent profile analysis was performed. Subsequently, logistic regression analysis was performed. Finally, mediation analysis was applied. The *p*-values lower than 0.05 are considered to be statistically significant (Bagozzi and Yi, 2012; Byrne, 2013; Kline, 2016; Tabachnick and Fidell, 2014).

Internal consistency was evaluated using JASP: Cronbach’s *α* and McDonald’s *ω*. Descriptive statistics and Pearson correlations were computed for composite variables using SPSS. To identify climate-belief profiles, we conducted LPA on the three climate-belief indicators, using JAMOVI which evaluated solutions with increasing numbers of profiles and alternative parameterizations; solutions with more than two profiles were not retained because they produced non-convergent or inadmissible solutions given the limited number of indicators, and/or resulted in very small classes. Model selection prioritized BIC, interpretability, and minimum class size. Next, we estimated a binary logistic regression predicting membership in the Higher Endorsement class (coded 1) versus the Lower Endorsement class (coded 0) from the seven science-belief items. Variance inflation factors (VIFs) were used to assess multicollinearity. Finally, mediation analyses were conducted using path modeling in JAMOVI with attitudes toward science as the mediator between personality traits and climate-change attitudes. Indirect effects were tested with normal-theory *z* tests and 95% confidence intervals.

## 3 Results

### 3.1 Reliability and descriptive statistics

Internal consistency of the key scales was acceptable to good for climate-change beliefs and attitudes toward science. For personality, the short-form nature of the BFI-2-XS produced modest reliabilities; only traits with *α* and *ω* ≥ 0.60 were retained for subsequent analyses (see Table 3).

**Table 3:** Internal consistency of study scales.

Scale (possible range)	*α*	*ω*	Interpretation
Climate-change beliefs (1–5)	0.825	0.827	Good
Attitudes toward science (1–5)	0.839	0.842	Good
Neuroticism (1–5)	0.718	0.720	Acceptable
Extraversion (1–5)	0.686	0.693	Acceptable
Conscientiousness (1–5)	0.640	0.640	Acceptable
Open-mindedness (1–5)	0.475	0.485	Not acceptable (excluded)
Agreeableness (1–5)	0.535	0.552	Not acceptable (excluded)

Descriptive statistics and correlations for retained study variables are reported in Table 4 (higher values indicate stronger endorsement).

**Table 4:** Descriptive statistics and Pearson correlations among the study variables.

Variable (range)	Mean	SD	1	2	3	4
1. Climate-change attitudes (1–5)	3.81	0.801	—			
2. Attitudes toward science (1–5)	3.53	0.707	0.363***	—		
3. Neuroticism (1–5)	2.70	0.918	−0.039	−0.043	—	
4. Conscientiousness (1–5)	3.53	0.875	0.079*	0.124***	−0.545***	—
5. Extraversion (1–5)	3.12	0.816	0.010	0.178***	−0.504***	0.473***

^*^p < 0.05; ^**^p < 0.01; ^***^p < 0.001.

Climate-change attitudes were positively correlated with attitudes toward science (*r* = 0.36, *p* < 0.001) and modestly with conscientiousness (*r* = 0.08, *p* = 0.05). Attitudes toward science were positively correlated with conscientiousness (*r* = 0.12, *p* < 0.001) and extraversion (*r* = 0.18, *p* < 0.001).

### 3.2 Latent profile analysis of climate-change beliefs

Subsequently, the Latent Profile Analysis (LPA) was conducted to identify subgroups within the sample based on their responses to three indicators related to climate change beliefs: (1) “Climate change is real,” (2) “Reason is human activity,” and (3) “Consequences will be dangerous.” We conducted LPA with complete data (*n* = 930).

Several models were compared using tidyLPA. An analytic hierarchy process utilizing multiple fit indices (AIC, AWE, BIC, CLC, and KIC), following the methodology outlined by Akogul and Erisoglu (2017), confirmed that the best-supported and interpretable solution was a two-class model.

Table 5 presents the fit indices for admissible latent profile solution. The overall fit statistics indicated that it provided the most parsimonious fit compared to the other models: Log-Likelihood: −3097; AIC: 6221; BIC: 6283; Entropy: 0.240. Despite the entropy value of 0.240, which indicates moderate classification accuracy and some overlap between the classes, the model still effectively differentiates between the two classes based on the variables assessed.

**Table 5:** Fit indices for admissible latent profile solution (complete cases, *n* = 930).

Model parameterization	Profiles	LogLik	AIC	BIC	Entropy	Notes
Model 3 (tidyLPA)	2	−3097	6221	6283	0.240	Selected (best BIC among admissible models)

Two latent classes were identified based on attitudes toward climate change. Class 1 (Lower Endorsement; *n* = 260, 29.2%) showed lower endorsement that climate change is real, that it is mainly human-caused, and that consequences will be dangerous. Class 2 (Higher Endorsement; *n* = 670, 70.8%) showed consistently high endorsement across the three indicators: higher means for all indicators, reflecting stronger beliefs in the reality of climate change, its human causes, and its severe consequences (see Table 6).

**Table 6:** Class-specific means and variability for climate-belief indicators.

Indicator (1–5)	Class 1 *M* (SE)	Class 1 SD	Class 2 *M* (SE)	Class 2 SD
Climate change is real	2.80 (0.07)	0.65	4.26 (0.03)	0.65
Reason is human activity	2.78 (0.05)	0.79	4.03 (0.04)	0.79
Consequences will be dangerous	2.91 (0.06)	0.64	4.30 (0.03)	0.64

**Figure 1:**
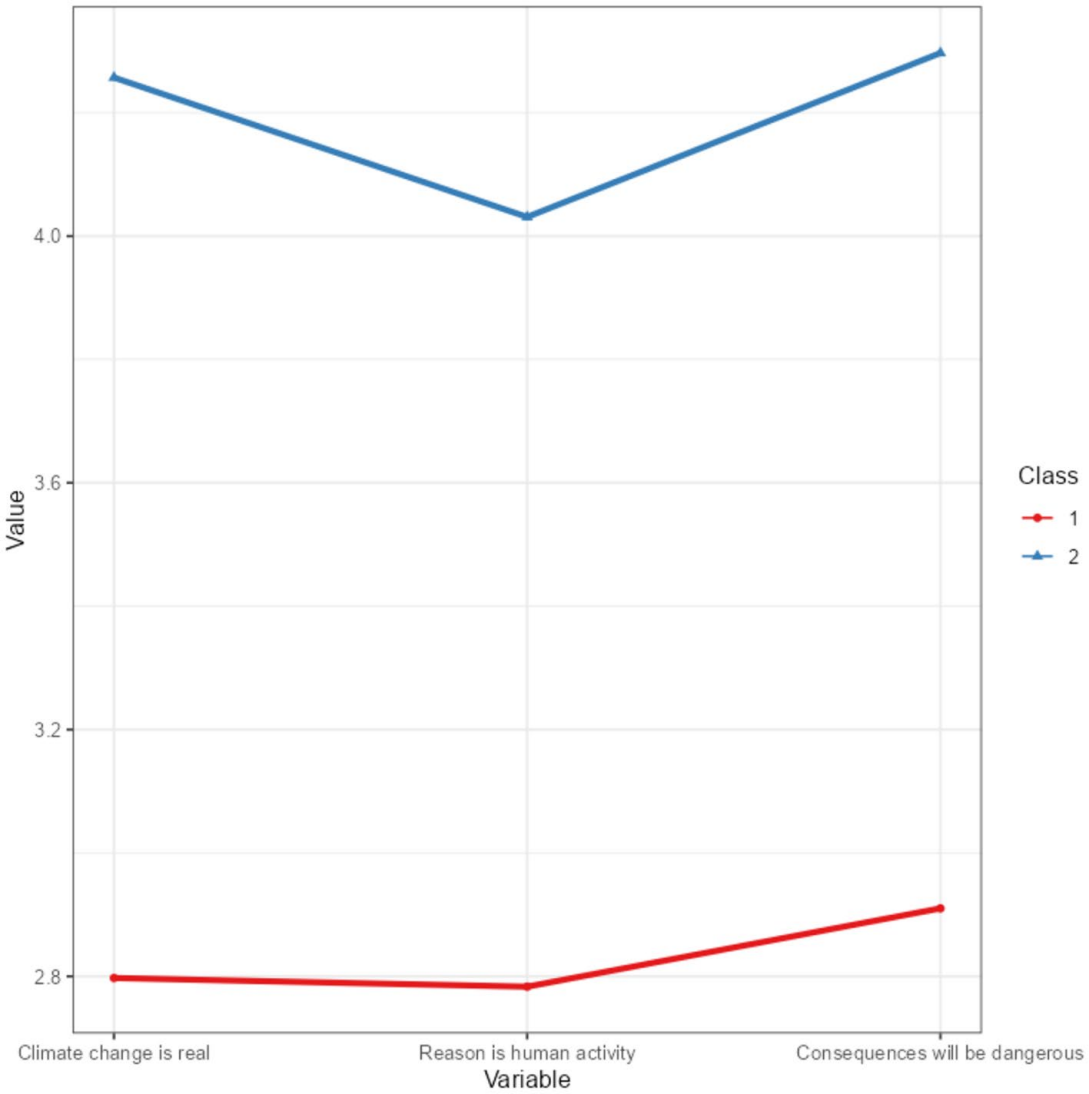
Climate-belief profiles across the two latent classes (line plot).

Class 1 (Lower Endorsement Group) shows a moderate level of endorsement of climate change reality (*M* = 2.797), human causes (*M* = 2.783), and dangerous consequences (*M* = 2.910). Class 2 (Higher Endorsement Group) strongly endorses the belief that climate change is real (*M* = 4.257), climate change is caused by human activity (*M* = 4.031), and climate change will have dangerous consequences (*M* = 4.297). Figure 1 presents line plot of the attitudes toward climate change in different classes.

Thus, the LPA revealed two distinct classes with differing levels of belief and endorsement regarding climate change. Class 1, representing approximately one-third of the sample, showed lower levels of belief in the reality, human causes, and consequences of climate change, while Class 2, constituting the majority of the sample, demonstrated strong endorsement of these beliefs.

### 3.3 Science-belief predictors of class membership

Next, we estimated a binary logistic regression predicting membership in the Higher Endorsement class (Class 2 = 1) relative to the Lower Endorsement class (Class 1 = 0) from seven science-belief items (*n* = 652 complete cases). The predictors included perceptions of science’s role in solving problems, its impact on lives, the scope of research, the necessity of research even when not immediately applicable, state funding for research, the role of science in political decision-making, and the balance of benefits and risks of science. The data provided insights into the significance of each predictor in determining class membership. The model fit was significant, *χ*^2^ (7) = 78.7, *p* < 0.001.

Collinearity diagnostics indicated no multicollinearity concerns (all VIFs <2). Tolerance values ranged from 0.622 to 0.859, which are acceptable thresholds, confirming that multicollinearity was not a significant issue in this analysis.

The Akaike Information Criterion (AIC) was 770, the Deviance was 758, and the Bayesian Information Criterion (BIC) was 806. These values suggest that the model provides a good fit to the data, with low AIC and BIC values indicating better model parsimony. Additionally, the overall model fit was evaluated with a Chi-square test, producing *χ*^2^ (7) = 78.7, *p* < 0.001, indicating a significantly better fit compared to the null model. Therefore, the model, as a whole, reliably differentiates between the two classes.

Next, the *R*^2^ values, including McFadden’s *R*^2^ (0.094), Cox & Snell’s *R*^2^ (0.114), Nagelkerke’s *R*^2^ (0.157), and Tjur’s *R*^2^ (0.122), indicated that the model explains a modest amount of variance in latent class membership, suggesting that while the model is statistically significant, the predictors may have a limited explanatory power.

The omnibus likelihood ratio tests revealed that attitudes “Science can solve any problem”, “There should be no limits on what can be studied” and “Research should be funded by the state” as predictors were statistically significant, with *p*-values of 0.029, <0.001, and 0.018, respectively, indicating that these predictors were important for distinguishing between latent classes. The results of the omnibus likelihood ratio tests for each predictor are displayed in Table 7.

**Table 7:** Omnibus likelihood ratio tests.

Variable	*χ* ^2^	d*f*	*p*
Science can solve any problem	4.742	1	0.029
Science improves people’s lives	0.949	1	0.330
There should be no limits on what can be studied	15.004	1	<0.001
Research is necessary, even if it may not be immediately applicable	0.004	1	0.947
Research should be funded by the state	5.557	1	0.018
Political decisions should be based on scientific findings	3.503	1	0.061
The benefits of science outweigh any possible negative effects	0.164	1	0.685

Table 8 presents the model coefficients, standard errors, odds ratios, and 95% confidence intervals for each predictor.

**Table 8:** Logistic regression coefficients predicting higher endorsement (class 2) vs. lower endorsement (class 1).

Predictor	*B*	SE	*z*	*p*	Odds ratio	95% CI (lower)	95% CI (upper)
Intercept	−4.3622	0.657	−6.6370	<0.001	0.0128	0.0035	0.0462
Science can solve any problem	0.2231	0.102	2.1832	0.029	1.2500	1.0231	1.5272
Science improves people’s lives	0.1296	0.133	0.9742	0.330	1.1384	0.8771	1.4776
No limits on what can be studied	0.4549	0.118	3.8425	<0.001	1.5760	1.2496	1.9876
Research is necessary, even if it may not be immediately applicable	−0.0093	0.140	−0.0662	0.947	0.9908	0.7527	1.3041
Research should be funded by the state	0.2991	0.127	2.3468	0.019	1.3486	1.0505	1.7312
Political decisions should be based on scientific findings	0.2342	0.125	1.8732	0.061	1.2639	0.9892	1.6147
The benefits of science outweigh any possible negative effects	0.0507	0.125	0.4055	0.685	1.0520	0.8232	1.3444

The analysis revealed that the belief “Science can solve any problem” as a predictor was statistically significant (*p* = 0.029). An increase in the belief that science can solve any problem increased the odds of belonging to Class 2 by 25% [OR = 1.250, 95% CI (1.023, 1.527)]. Similarly, the belief “There should be no limits on what can be studied” as a predictor was also significant (*p* < 0.001). An increase in the belief that there are no limits on what can be studied increased the odds of Class 2 membership by 57.6% [OR = 1.576, 95% CI (1.250, 1.988)]. The belief “Research should be funded by the state” as predictor was also significant (*p* = 0.019) and also had a positive effect, with an odds ratio of 1.35 [95% CI (1.05, 1.73)]. Supporting state-funded research increased the odds of belonging to Class 2 by approximately 34.9%.

However, the beliefs “Science improves people’s lives”, “Research is necessary even if not immediately applicable”, “Political decisions should be based on scientific findings”, and “The benefits of science outweigh any possible negative effects” were not statistically significant predictors in the model, which means they did not significantly differentiate between class membership.

Thus, the logistic regression model effectively differentiated between class memberships based on several beliefs about science. Significant predictors such as “Science can solve any problem,” “No limits on what can be studied,” and “Research should be funded by the state” provide critical insights into the factors contributing to class membership. The non-significant predictors highlight areas where beliefs may not strongly influence class differentiation, suggesting that not all positive attitudes about science directly impact class membership.

### 3.4 Personality, attitudes toward science, and attitudes toward climate change

Furthermore, we tested a mediation model in which conscientiousness, extraversion, and neuroticism predicted climate-change attitudes directly and indirectly via attitudes toward science. Table 9 summarizes direct, indirect, and total effects.

**Table 9:** Direct, indirect, and total effects on attitudes toward climate change.

Predictor	Effect type	*B*	Std. error	*z*-value	*p*-value	95% CI (lower, upper)
Conscientiousness	Direct	0.088	0.045	1.939	0.053	0.00096, 0.177
Extraversion	Direct	−0.133	0.048	−2.791	0.005	−0.227, −0.040
Neuroticism	Direct	−0.065	0.044	−1.472	0.141	−0.153, 0.022
Conscientiousness	Indirect via attitudes toward science	0.046	0.020	2.371	0.018	0.008, 0.085
Extraversion	Indirect via attitudes toward science	0.082	0.021	3.882	<0.001	0.041, 0.124
Neuroticism	Indirect via attitudes toward science	0.039	0.019	2.031	0.042	0.001, 0.076
Conscientiousness	Total	0.135	0.048	2.801	0.005	0.040, 0.229
Extraversion	Total	−0.051	0.050	−1.015	0.310	−0.149, 0.047
Neuroticism	Total	−0.027	0.047	−0.568	0.570	−0.119, 0.065

For Conscientiousness, the direct effect on attitudes toward climate change was found to be marginally significant, *b* = 0.088, SE = 0.045, *z* = 1.939, *p* = 0.053, with a 95% confidence interval (CI) ranging from 0.00096 to 0.1770. This indicates a positive but weak relationship, suggesting a potential direct effect of Conscientiousness on climate change attitudes.

Extraversion had a significant negative direct effect on attitudes toward climate change, *b* = −0.133, SE = 0.048, *z* = −2.791, *p* = 0.005, 95% CI (−0.227, −0.040). This result suggests that individuals with higher levels of Extraversion may have less favorable attitudes toward climate change.

For Neuroticism, no significant direct effect was observed on attitudes toward climate change, *b* = −0.065, SE = 0.044, *z* = −1.472, *p* = 0.141, 95% CI (−0.153, 0.022). Thus, Neuroticism does not appear to directly contribute to climate change attitudes.

The analysis of indirect effects via attitudes toward science showed that Conscientiousness revealed a significant positive indirect effect on attitudes toward climate change through attitudes toward science, *b* = 0.046, SE = 0.020, *z* = 2.371, *p* = 0.018, 95% CI (0.008, 0.085). This indicates that Conscientiousness can positively affect attitudes toward climate change by enhancing attitudes toward science.

Extraversion also had a significant positive indirect effect through attitudes toward science, *b* = 0.082, SE = 0.021, *z* = 3.882, *p* < 0.001, 95% CI (0.041, 0.124). Despite its negative direct effect, the indirect pathway through science attitudes moderates some of the negative contribution.

Neuroticism’s indirect effect on climate change attitudes via attitudes toward science was significant as well, *b* = 0.039, SE = 0.019, *z* = 2.031, *p* = 0.042, 95% CI (0.001, 0.076), suggesting that while Neuroticism did not have a direct impact, its impact on climate change attitudes could be mediated through positive attitudes toward science.

The total effect of Conscientiousness on attitudes toward climate change was significant, *b* = 0.135, SE = 0.048, *z* = 2.801, *p* = 0.005, 95% CI (0.040, 0.229). This suggests that Conscientiousness contributes to attitudes toward climate change through both direct and indirect pathways.

Extraversion’s total effect on climate change attitudes was not significant, *b* = −0.051, SE = 0.050, *z* = −1.015, *p* = 0.310, 95% CI (−0.149, 0.047). This result implies that the direct and indirect effects may cancel each other out.

Neuroticism’s total effect on climate change attitudes was also non-significant, *b* = −0.027, SE = 0.047, *z* = −0.568, *p* = 0.570, 95% CI (−0.119, 0.065).

The path coefficients analysis also showed that attitudes toward science had a significant positive effect on attitudes toward climate change, *b* = 0.383, SE = 0.034, *z* = 11.339, *p* < 0.001, 95% CI (0.317, 0.449). This indicates that fostering positive attitudes toward science is a key factor in improving attitudes toward climate change.

Conscientiousness had a positive effect on attitudes toward science, *b* = 0.121, SE = 0.050, *z* = 2.426, *p* = 0.015, 95% CI (0.023, 0.219), suggesting that conscientious individuals are more likely to have favorable attitudes toward science.

Extraversion was positively associated with attitudes toward science, *b* = 0.214, SE = 0.052, *z* = 4.114, *p* < 0.001, 95% CI (0.112, 0.317). This result shows that more extroverted individuals tend to have more positive attitudes toward science, which can in turn progressively contribute to their attitudes toward climate change.

Neuroticism also had a significant positive effect on attitudes toward science, *b* = 0.101, SE = 0.049, *z* = 2.065, *p* = 0.039, 95% CI (0.005, 0.197). This suggests that neurotic individuals may hold positive views toward science, which subsequently could contribute to their views on climate change.

**Figure 2:**
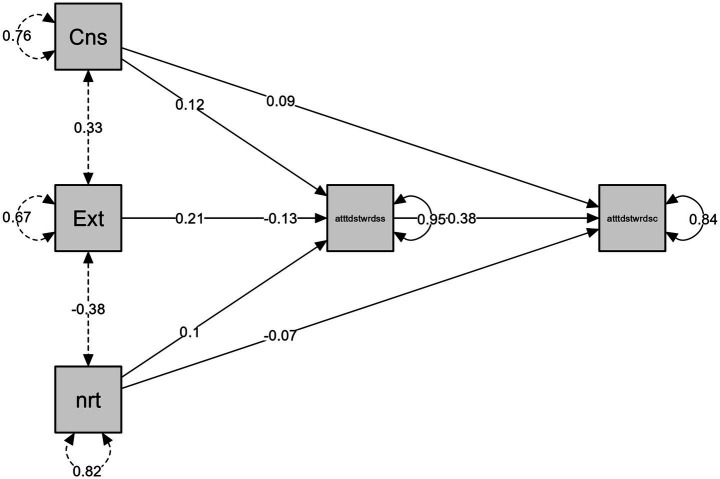
Mediation analysis path plot: the links between conscientiousness (Cns), extraversion (Ext), neuroticism (nrt) to attitudes toward climate change (atttdstwrdsc) through attitudes toward science (atttdstwrdss).

To summarize, the findings highlight the complexity of the relationships between personality traits, attitudes toward science, and attitudes toward climate change. Conscientiousness positively contributes to climate change attitudes directly and indirectly through science attitudes. While Extraversion has a negative direct effect, its indirect effect through science attitudes is positive, implying that enhancing scientific engagement can counterbalance negative attitudes toward climate change. Neuroticism did not show a direct effect on climate change attitudes, but the positive indirect effect through attitudes toward science suggests the importance of promoting scientific literacy among those with higher negative emotionality (see Figure 2).

## 4 Discussion

Based on the results of the latent profile analysis (LPA), logistic regression, and mediation analysis, this study provided evidence on the relationship between personality traits, attitudes toward science, and climate change beliefs. Across analyses, attitudes toward science emerged as a central factor: they were positively correlated with climate-change attitudes, differentiated profile membership, and mediated links between personality traits and climate-change attitudes.

The two-class LPA solution is consistent with segmentation research showing that publics often divide into groups that vary in acceptance of climate science and perceived risk. In Lithuania, most respondents fell into a Higher Endorsement class, but nearly one-third expressed lower endorsement of climate-change reality, anthropogenic causation, and risk. This split is practically meaningful: it indicates that even within a context where broad concern about climate exists, a sizable minority may be less receptive to standard climate messaging. Lithuanian public opinion data suggest that climate issues may compete with other salient concerns (e.g., security, economy), which can affect attention and motivation to engage with climate information (European Parliament Research Service, 2025; Nordic Council of Ministers Office in Lithuania, 2025). On the whole, the findings of this study align with prior studies that suggest the public can be divided into subgroups based on their level of climate change belief and skepticism (Metag and Schäfer, 2018). Understanding these different segments allows researchers and communicators to tailor messages that specifically address the unique needs and misconceptions of each group, which can help increase engagement and reduce skepticism (Scheufele, 2018).

Science attitudes that implied “confidence in science as an unlimited problem solver” and “support for unrestricted inquiry” were the strongest predictors of belonging to the Higher Endorsement class. These beliefs likely reflect broader epistemic trust and perceptions of science as legitimate and capable, which are known to support acceptance of scientific consensus on contested issues (Fischhoff, 2019; Kappel and Holmen, 2019). Notably, some pro-science beliefs (e.g., “science improves lives”) did not differentiate classes, suggesting that generalized positivity toward science is not always sufficient; rather, specific beliefs about science’s scope, authority, and role in society may matter more for climate acceptance.

The findings suggest that individuals with a stronger belief in the problem-solving power of science are 25% more likely to belong to the group that strongly endorses climate change beliefs. Next, those who believe there should be no limits on scientific research are 57.6% more likely to belong to the group that strongly endorses climate change beliefs. Third, supporting state-funded research increased the odds of belonging to the group that strongly endorses climate change beliefs by 34.9%. This supports the argument that science communication is very important (Kappel and Holmen, 2019) and promoting the importance of scientific inquiry could be crucial for contributing to public opinions about climate change.

The mediation analysis results provide additional insights into the links between personality traits, attitudes toward science, and attitudes toward climate change. Conscientiousness exhibited a positive total effect on climate-change attitudes, partly through attitudes toward science, consistent with the idea that conscientious individuals prefer orderly, evidence-based approaches and may be more responsive to responsibility-framed sustainability messages (Weiler et al., 2012). Extraversion showed a negative direct association with climate-change attitudes, but a positive indirect association through science attitudes, indicating that extraverted individuals may benefit from engagement-oriented science communication that leverages social interaction and collective action framing. This result highlights the importance of fostering curiosity and excitement about science, especially among extroverted individuals, to increase climate engagement (Hirsh et al., 2012). Neuroticism did not have a significant total effect, yet the indirect path through science attitudes was positive, implying that strengthening trust in science may be particularly important for audiences experiencing higher uncertainty or threat sensitivity. This implies that individuals with higher Neuroticism might benefit from interventions that improve their attitudes toward science, potentially making them more receptive to climate change messages. The role of science narratives in reducing anxiety and increasing engagement might be important for these individuals (Bilandzic et al., 2020).

Lithuania has a distinct post-Soviet institutional history and evolving media environment, which may shape public trust in institutions, perceptions of expertise, and engagement with global risks. Therefore, segmentation results should be interpreted in light of local patterns of institutional trust, political polarization, and media consumption. At the same time, the overall structure, including a high-belief majority and a skeptical minority linked to weaker pro-science orientations, resembles patterns observed in other European contexts, suggesting that the identified mechanisms may generalize beyond Lithuania while differing in magnitude.

### 4.1 Implications

The findings suggest several key considerations for science communication. Given the two distinct classes, communication strategies should be adapted to address the different levels of endorsement regarding climate change. For the skeptical group (Class 1), communication should focus on addressing misconceptions, potentially leveraging narratives to create emotional connections to climate issues (Bilandzic et al., 2020; Burns and Medvecky, 2018). For Class 2, messages could emphasize actions and empower individuals who already believe in climate change to take further steps.

The mediation analysis underlines the importance of considering personality traits in communication strategies. For extroverted individuals, fostering engagement with science through interactive and community-based initiatives may help bridge their lower direct belief in climate issues (Olsacher et al., 2023). For neurotic individuals, empathetic approaches that provide reassurance and emphasize the positive role of science could be effective (Bilandzic et al., 2020).

The results revealed that attitudes toward science were a significant mediator. Therefore, enhancing public perceptions of science can play a critical role in contributing to climate change attitudes, especially for individuals less inclined to believe in climate change directly. Evidence-based science communication practices, such as making the content relatable and addressing misconceptions, are essential (Fischhoff, 2019; Jensen and Gerber, 2019).

The logistic regression findings underscore the importance of fostering support for scientific research, both in its scope and funding. Emphasizing the value of unrestricted scientific exploration and the role of government in supporting research can be crucial in shifting public beliefs toward climate change (Kappel and Holmen, 2019).

To sum up, the findings support a segmentation-first approach to climate communication. For the Lower Endorsement class, communicators may need to prioritize trust-building, address misconceptions, and provide locally relevant narratives that connect climate risks and solutions to everyday Lithuanian experiences (e.g., agriculture, forests, and extreme weather impacts). For the Higher Endorsement class, messages may focus on efficacy, concrete behavioral pathways, and collective action opportunities. Because attitudes toward science were a robust mediator and predictor, interventions that strengthen perceptions of science as legitimate, transparent, and socially responsive may indirectly increase acceptance of climate science. Finally, incorporating personality-informed tailoring, such as action- and community-focused formats for extraverted audiences, may enhance engagement (Hirsh et al., 2012; Olsacher et al., 2023).

### 4.2 Limitations and future research

Several limitations should be considered. First, the design is cross-sectional; causal claims cannot be made and temporal ordering in mediation models is theoretical rather than empirically established. Second, climate-change beliefs were measured with three indicators, which may not capture the full multidimensionality of climate attitudes (e.g., policy preferences, emotions, and behavioral intentions). Future studies should use broader item batteries and test measurement models explicitly. Third, the two-class LPA solution showed low entropy, indicating classification uncertainty and overlap between classes; results should therefore be interpreted as probabilistic segmentation. Fourth, list-wise deletion reduced sample sizes in some analyses, potentially affecting power and representativeness. Fifth, only three Big Five domains were retained due to limited internal consistency in the short-form personality measure; future work should employ longer validated inventories or latent-variable modeling to improve measurement precision.

## 5 Conclusion

Audience segmentation reveals actionable heterogeneity in climate-change beliefs in Lithuania. Attitudes toward science appear to be a key differentiator between skeptical and non-skeptical groups and a pathway linking personality traits to climate-change attitudes. These findings support communication strategies that combine audience segmentation with interventions designed to strengthen trust in and perceived relevance of science.

## Data Availability

The original contributions presented in the study are included in the article/supplementary material, further inquiries can be directed to the corresponding author.
